# Agricultural habitat use and selection by a sedentary bird over its annual life cycle in a crop-depredation context

**DOI:** 10.1186/s40462-024-00462-0

**Published:** 2024-03-29

**Authors:** Rémi Chambon, Jean-Marc Paillisson, Jérôme Fournier-Sowinski, Sébastien Dugravot

**Affiliations:** 1 UMR 7208 - BOREA, MNHN, CNRS, UPMC, IRD, UC, UA, Université de Rennes, Rennes, France; 2 UMR 6553 - ECOBIO, CNRS, Université de Rennes, Rennes, France; 3 UMR 7204 - CESCO, MNHN, CNRS, Station marine de Concarneau, Concarneau, France

**Keywords:** *Corvus monedula*, Crop damage, Foraging duration, GPS telemetry, Land use, Occurrence distribution, Resource selection

## Abstract

**Background:**

Modern agriculture has undoubtedly led to increasing wildlife-human conflicts, notably concerning bird damage in productive and attractive crops during some parts of the annual cycle. This issue requires utmost attention for sedentary birds that may impact agricultural crops at any stage of their annual life cycle. Reducing bird-human conflicts requires a better understanding of the relationship between bird foraging activity and the characteristics of agricultural areas, notably with respect to changes in food-resource availability and crop sensitivity across the year.

**Methods:**

We explored how GPS-tagged adult male western jackdaws– sedentary corvids– utilize agricultural areas throughout their annual cycle, in a context of crop depredation. More precisely, we described their daily occurrence distribution and the extent of habitat use and selection consistency with respect to landscape composition across time.

**Results:**

Jackdaws moved in the close agricultural surroundings of their urban nesting place over the year (< 2.5 km from the nest, on average). Daily occurrence distributions were restricted (< 2.2 km^2^), relatively centered on the nesting locality (distance between the daily occurrence centroid and the nest < 0.9 km), and rather spatially stable during each annual life-cycle period (overlap range: 63.4–76.1%). Their foraging patterns highlighted that they fed mainly in grasslands all year round, and foraged complementarily and opportunistically in maize (during sowing– coinciding with the first stages of the birds’ breeding period) and cereal crops (during harvesting– their post-fledging period).

**Conclusions:**

Our findings demonstrate the very limited space use by breeding male jackdaws which foraged preferentially in grasslands. We call for future investigations in other agricultural contexts and also considering non-breeders for extrapolation purposes.

**Supplementary Information:**

The online version contains supplementary material available at 10.1186/s40462-024-00462-0.

## Background

It is now well acknowledged that modern agriculture (intensive agriculture centered on monocropping) combined with habitat loss (the conversion of natural lands to agriculture) are the major causes of wildlife decline, notably of bird species [1–4]. On the other hand, productive crops in agricultural landscapes are highly attractive to feeding birds including cranes [5], waterfowl (notably geese [6, 7]), and other species classified as bird pests due to the substantial crop damage they cause [8]. The combination of the increasing use of agricultural crops and the population growth of some of these birds is likely to exacerbate conflicts with farmers [9, 10]. Reducing bird-human conflicts is a crucial challenge that cannot be successfully met without a robust understanding of the relationships between bird foraging behavior and characteristics of agricultural land [8, 11].

Problematic birds generally have broad environmental tolerance and are very opportunistic: they can exploit supplementary food resources not monopolized by other species or resources that other species are not able to exploit [12]. Birds are also highly mobile, and the way they make use of space has long been a challenging issue [13]. Even if the development of satellite tracking technology now allows studying the movement of free-flying birds [13, 14], the spatial use of agricultural areas by undesirable birds is still limited to a few species (e.g. [15, 16]). For example, biotelemetry studies account for only 5% of existing literature on the foraging activity of birds causing crop depredation (82 relevant articles on this issue was found on the Web of Science in October 2023 using the following search terms: bird AND forag* AND crop damag*).

The selection of foraging habitats is central for birds throughout their annual cycle and is not restricted to breeding (when food needs peak): sedentary species have to cope with possible depletion in food resources (and increased competition) during winter [17, 18]. Bird foraging patterns in agricultural areas are mainly influenced by landscape composition and farming practices [19, 20]. Individuals are expected to forage mainly in higher-quality habitats, that provide more abundant and accessible food resources [21, 22]. For example, omnivorous and opportunistic birds mainly feed in fields with a limited crop cover to access soil prey (e.g. common gulls *Larus canus* in [23]). In addition, birds may use and revisit specific areas when food resources are not only profitable, but also predictable across space and time [24, 25]. However, food resources typically vary greatly in space and time in agricultural areas with respect to farming practices, and foraging habitat use and selection patterns by birds are supposed to vary accordingly.

The Western jackdaw (*Corvus monedula*) is a cavity-nesting, semi-colonial, synurbanistic corvid [26–29]. Like other corvids, it prefers man-made landscapes, notably in western Europe, and is well adapted to living in both urban and rural habitats due to its high ecological adaptability [30–32]. Jackdaws are opportunistic omnivores that mainly feed on seeds and invertebrate prey sought for in agricultural settings (mainly maize and cereal crops, but also some vegetable crops) and other habitats such as natural grasslands [33–36] (Table S1). In Europe, damage to maize and cereal crops by birds (mainly cranes, geese, pigeons, starlings and corvids) is primarily related to sowing and young plants ([8, 37] and references therein), but also to standing cereals just before harvesting for jackdaws [36] (Fig. S1). The recent expansion and demographic increase of the species in France [38, 39] combined to its opportunistic feeding regime and sedentary behavior has raised growing concerns from farmers. For example, in Brittany (western France), government services received 439–1496 complaint cases from farmers related to crop depredation by jackdaws in 2020–2022 (69–85% focusing on maize), valued at around 1.2–2.8 million euros of damage annually [40]. Finally, even if habitat use by jackdaws can be partly and indirectly inferred from diet information, it does not provide extensive information on their foraging range, notably their fine-scaled use of space and daily time budget. Given their strong nest attendance all year round [26], adult jackdaws are likely central-place foragers and are supposed to be geographically constrained when foraging. This means that their feeding areas are probably limited by how far they can fly from their urban nesting places, which they defend both during the breeding and non-breeding periods, and depending on their energetic needs and food availability.

The aim of this study was to address the issue of space use by adult jackdaws in a crop depredated region. More precisely, we studied how GPS-tagged adult male jackdaws moved in a Brittany agricultural area over their annual cycle by quantifying seasonal occurrence distributions, and foraging habitat use and selection with respect to the agricultural landscape composition. We attached great importance to addressing this issue relatively to the season (see also [8, 17]) and the related spatio-temporal dynamics of environmental conditions (including food-resource availability and crop sensitivity; [8]), something which remains understudied (but see [15, 16]). Given the reasoning above, jackdaws are expected to spend more time feeding on the most readily available resources across the year. More specifically, since conflicts with farmers mainly arise from damage on maize seedlings and cereals just before harvest, jackdaws were expected to largely exploit these crops during these specific periods. Those food resources being ephemeral, we expected temporal variation in habitat use and selection in line with seasonality in habitat cover and bird needs (breeding *versus* non breeding periods). We jointly studied the importance of grasslands as possible additional feeding areas. Our study provides a basic understanding of the relationships between foraging activities by jackdaws and the characteristics of agricultural landscapes, which are key to applied issues.

## Methods

### Study area and fieldwork

**Fig. 1:**
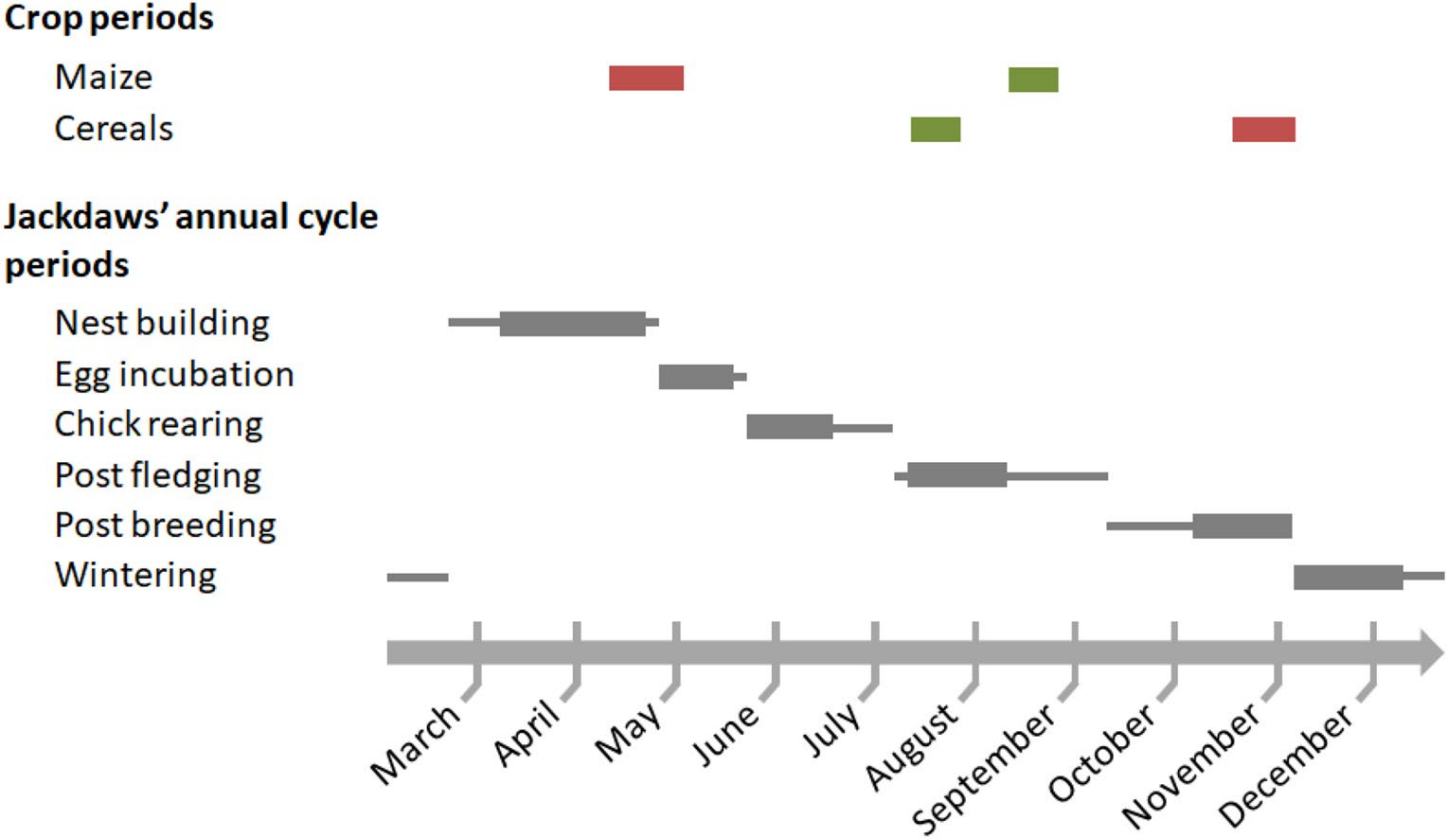
Peak periods of sowing and harvesting matching with tracking periods (gray rectangles) throughout the annual life cycle of jackdaws (thick gray lines as biological periods). We focused on maize and cereal (wheat and barley, mostly) which are the main depredated crops in our study area [40]. Red and green, peak periods of sowing and harvesting, respectively

The study of bird movements was conducted in southern Brittany in 2021 and 2022, in the Quimperlé region (47.866° N, 3.550° W), where crop damage has been frequently reported in the past 15 years, probably in link with the increase in jackdaws’ population size observed in Brittany [36, 41]. This area is representative of farming in western France [42]. A large proportion of the agricultural landscape is cropped with maize (around 30%) and cereals (mainly wheat and barley, 29%); natural and cultivated grasslands represent 33%, and the remaining 8% are other various crops including vegetables (green bean, green pea, potato, carrot, cabbage), oilseed (sunflower), fruit trees, fallow and others, mainly aromatic plants (data source: habitat map in Brittany from the Conservatoire National Botanique de Brest, combined with 2021 and 2022 crop maps (the Registre Parcellaire Graphique) from the Institut National de l’Information Géographique et Forestière [43]). We refer thereafter to the sowing and harvesting periods in these two years based on data from the Chambre Régionale d’Agriculture de Bretagne (a regional administrative authority) and personal field observations to interpret the foraging activity patterns of jackdaws throughout their annual cycle (Fig. 1).

Jackdaws were opportunistically caught on four days (May 20 and 21, then July 7 and 8) in 2021 using six large cage traps (length × width × height: 3 × 2 × 2 m). These cages were deployed on fields by trappers under the supervision of the Direction Départementale des Territoires et de la Mer (a local administrative authority) during a control campaign in response to crop damage (under derogations due to the protected status of the species). We equipped 20 birds, including 13 individuals in their breeding age (adults, i.e. > one year old; [26, 44]) and 7 immature (one-year-old) birds at the time of capture (age estimated based on plumage maturation at capture [45]). We attached a solar-powered GPS-GSM transmitter (OrniTrack-10, Ornitela, UAB, Lithuania; 10 g, i.e. < 5% of individual body mass) on their back with a teflon wing harness. They were also banded with an individually numbered metal ring from the Centre de Recherches sur la Biologie des Populations d’Oiseaux, plus a numbered blue plastic ring on their two tibiotarsi. All birds were males (molecularly sexed from feathers; [46]), since we equipped the heaviest individuals to allow them to bear the tag and because females were still incubating during some capture sessions [45].

To prevent battery depletion, the tags were programmed to intermittently record locations based on 6-day periods. In other words, acquisition and resting 6-day periods alternated all year round. During the acquisition periods, locations were recorded every five minutes between sunrise and sunset each day (local times estimated using www.sunrise-and-sunset.com). Data recording by some devices was occasionally suspended during the acquisition period to preserve the battery level. The tags were programmed to initiate data collection five days after deployment in 2021, time for the birds to adapt to the equipment. No location was collected from mid-December 2021 to early February 2022 due to restricted sunlight and the risk of battery damage, and the still active tags were switched-off from the end of October 2022.

For sample size and biological reasons, we only focused on the movement patterns of breeding adult males. Thus, for the individuals equipped when immature in 2021, GPS data used in the present analyses were only considered from the spring 2022 (see more details below), when they all nested as adults (see below). Furthermore, even if both pair partners invest into provisioning and parental care during breeding, their roles differ [44]. Males indeed predominantly supply chicks and their female partner with food, while females incubate and rarely leave their nest before the chicks are 20 days old [27, 47].

GPS data were split into six tracking periods (also called ‘biological periods’ thereafter) according to the annual breeding cycle of the population. They partially covered (due to data availability) the breeding period, the post-fledging period (with some remaining parental care), and the non-breeding period. The breeding period was split into three standard periods: nest construction (10 March–20 April, both partners participate to this stage; [48]), egg incubation (27 April–14 May, female-specific task, males provision their mate with food; [27, 47]), and chick rearing (21 May–16 June, inconsistent contribution from the two partners, [27]). During the post-fledging period (10 July–8 August), fledglings are still provisioned by their parents for food but become progressively self-sufficient for foraging and live away from the family unit. Lastly, the non-breeding period was also partitioned into two periods: post breeding (5 October–3 November, no parental care) and wintering (6 November–6 December; no location was collected in the following winter weeks as previously explained). The timing of each biological period (Fig. 1) was based on field observations in the study area (fairly consistent with those found in existing literature: [36] and references therein), plus constraints due to GPS data availability. The number of tracking days (i.e. a combination of date and bird identity) per period is summarized in Table 1 (see also Table S2).

**Table 1:** Details of the tracking data from the equipped jackdaws over their annual life cycle

Annual cycle period	Number of year-birds (individuals)	Tracking days		Mean number of locations per tracking day ± SD
		Total	Mean ± SD per bird (range)	
Nest building	18 (18)	156	8.7 ± 4.6 (1–19)	150.0 ± 10.1
Incubation	14 (14)	61	4.4 ± 3.6 (1–11)	173.8 ± 3.7
Chick rearing	19 (16)	61	3.2 ± 2.3 (1–8)	187.2 ± 2.1
Post fledging	17 (14)	139	8.2 ± 4.5 (1–15)	179.0 ± 4.7
Post breeding	12 (12)	77	6.4 ± 2.4 (2–10)	129.0 ± 6.3
Wintering	9 (9)	38	4.2 ± 2.5 (1–8)	105.6 ± 4.3

Data recorded from 20 individuals (31 year-birds when the bird identity was associated with year; 532 tracking days totalling 84 192 GPS locations) in 2021 and 2022 are combined. Tracking day: a combination of date and bird identity. Details per bird are provided in Table S2

In our study area, jackdaws nest mostly in chimneys (and in wall and roof cavities to a lesser extent) in old urban neighborhoods [36]. The nest locations of the equipped birds were inferred from GPS data and validated by visual observations. Birds were found breeding in four close towns separated by an average 15 km (5–28 km; Table S2). Jackdaws were faithful to their nest site: 70% of the birds that nested in 2021 re-used the same nest in 2022, while the others were found breeding on close neighboring chimneys.

For all the data and analyses presented, tracking days with more than 5% of missing locations were discarded, and occasional missing locations from the remaining tracking days were interpolated using a continuous-time correlated random walk modeling approach [49].

### Descriptors of daily occurrence distributions

Daily occurrence distributions were assessed for each bird using a biased random bridge method [50, 51] on all locations available for each tracking day. This method provides 95% utilization distribution estimates taking the spatio-temporal autocorrelation issue for bird location and movement into account. In accordance to a recent review [52], this method is suitable for the calculation of the movement descriptors we used on a tracking day basis (i.e. for a limited period of time). This time unit is relevant and easy to interpret regarding the structure of our data and the present issue. Four complementary descriptors (Fig. S2) were calculated to describe potential variation in occurrence distributions among the six biological periods, considering the year-round nest attendance displayed by breeders [26]: (i) the area (km^2^) of each daily occurrence distribution, (ii) the mean spatial overlap ratio (%) of each daily occurrence distribution over the other daily occurrence distributions available for each bird within each annual cycle period (proxy of spatial fidelity of the daily activities), (iii) the distance between the centroid of each daily occurrence distribution and the nest site (km, measure of the centrality of the nest over the daily activities), and (iv) the distance between the farthest limit of each daily occurrence distribution and the nest site (km).

We explored the differences in these metrics over the annual cycle using separate Generalized Linear Mixed Models (GLMMs, with gamma error distribution and log link function; but we used a LMM for spatial fidelity). The biological period was the only fixed effect, and the identity of birds associated with year (namely a ‘year-bird’ grouping factor) was used as a random effect to take repeated measures into account (one datum per tracking day). In other words, each bird monitored in both years was considered as different individuals due to crop rotation and differences in habitat use experience between years. For each model, residuals were checked, the effect of the fixed term was tested (Wald chi-square test), pairwise post-hoc comparisons (considering Tukey-adjusted *p*-values) were performed, and both marginal and conditional pseudo R-squared (R^2^_m_ and R^2^_c_) were computed as a measure of the variance explained by the sole fixed effect and by both the fixed and random terms, respectively (Table S3).

### Foraging use and selection of agricultural habitats

Before exploring the issues of habitat use and selection, we filtered locations linked to foraging occasions in agricultural habitats. We used a Hidden Markov Model that accurately discriminates three distinct behaviour classes based on the distribution of smoothed speed and turning angle between consecutive GPS locations (behavioral path-segmentation model; [53, 54]): ‘stationary’ activity (rest and highly spatially restricted walk), short-distance movements (active walk), and long-distance movements (flight; see Fig. S2). Gamma and von Mises distributions were used for the speed and angle variables, respectively. The mean estimated speed for each behavior class (3.1, 14.2 and 78.8 m/min for stationary, short-distance and long-distance behaviors, respectively) was consistent with our expertise on jackdaw activities, and were robust to initial-value changes and pseudo-residual checking. Consequently, any location associated with a resting or walking behavior in an agricultural habitat (see full description above) was considered as a foraging occasion in agricultural land.

We explored the extent of foraging use of each agricultural land type (habitat) over time (i.e. according to bird needs, seasonality in habitat cover, and crop sensitivity). In practice, we compared the daily use probability of each main foraging habitat (grasslands, cereals and maize– 65%, 24% and 8%, respectively, of all foraging occasions combined, *n* = 21 188 occasions) over the annual life-cycle using separate GLMMs (binomial error distribution and logit link function). The biological period was used as the unique fixed effect, and the year-bird grouping factor was used as a random effect. Complementarily, we compared the daily time (min) spent by the birds foraging in each of these three foraging habitats over the annual life-cycle periods using separate GLMMs (gamma error distribution and log link function). The biological period was used as the unique fixed effect, and the year-bird grouping factor as a random effect. The duration of daily foraging by a bird in a given agricultural habitat was estimated as the total number of foraging locations recorded in this habitat type multiplied by five minutes (the GPS acquisition time lag), solely considering the tracking days when the bird foraged in the given habitat (i.e. foraging duration conditional on use of the focal habitat). For each GLMM, we checked residuals, tested the effect of the fixed term (Wald chi-square test), computed pairwise post-hoc comparisons (considering Tukey-adjusted *p*-values) and calculated R^2^_m_ and R^2^_c_ (Table S3).

Finally, we explored preferences in habitat use by jackdaws during each biological period using habitat selection functions (HSFs; [55]). In practice, we considered the three most visited foraging habitats, plus a fourth habitat (‘other’) grouping all other agricultural habitats that were less regularly utilized, and fitted a distinct HSF model for each biological period. For each recorded foraging location, ten locations were randomly generated within a circle centered on the nest location whose radius was the distance between the farthest limit of each daily occurrence distribution and the nest location (available and accessible space), according to the tracking day and tagged bird. The agricultural habitat associated to each random location was identified. HSFs were weighted GLMMs (binomial error distribution and logit link function; assigning weights of 1000 and 1 to random and recorded locations, respectively). Habitat type was used as the unique fixed effect, with the random nested effect of tracking day and year-bird identity (random intercept fixed with a large variance, following [56]). ‘Grasslands’ was used as the reference habitat to measure the relative strength of habitat selection [55], since this habitat is not critical from an economic point view regarding jackdaw-farmer conflicts, and also because it was frequently used over the annual cycle (see below). All the fitted HSFs were robust to k-fold cross-validation ([57]; 0.56 < Spearman’s rank correlation < 0.95; see Table S4).

All statistical analyses were conducted in R (v.4.2.1; [58]), mainly using the packages ”adehabitatLT”, ”adehabitatHR”, “amt”, “momentuHMM”, “lme4”, ”glmmTMB”, ”RVAideMemoire”, ”MuMIn”, ”emmeans”, and ”ggeffects”. The significance level was fixed at *α* = 0.05.

## Results

Following results came from 532 tracking days totaling 84 192 locations from the 20 tracked jackdaws (31 distinct year-birds) over two years (Tables 1 and S2). The number of tracking days varied according to the six biological periods, and quite naturally the number of locations per tracking day peaked during chick rearing and was lower during wintering in link with the photoperiod.

### Temporal variation of daily occurrence distributions

**Fig. 2:**
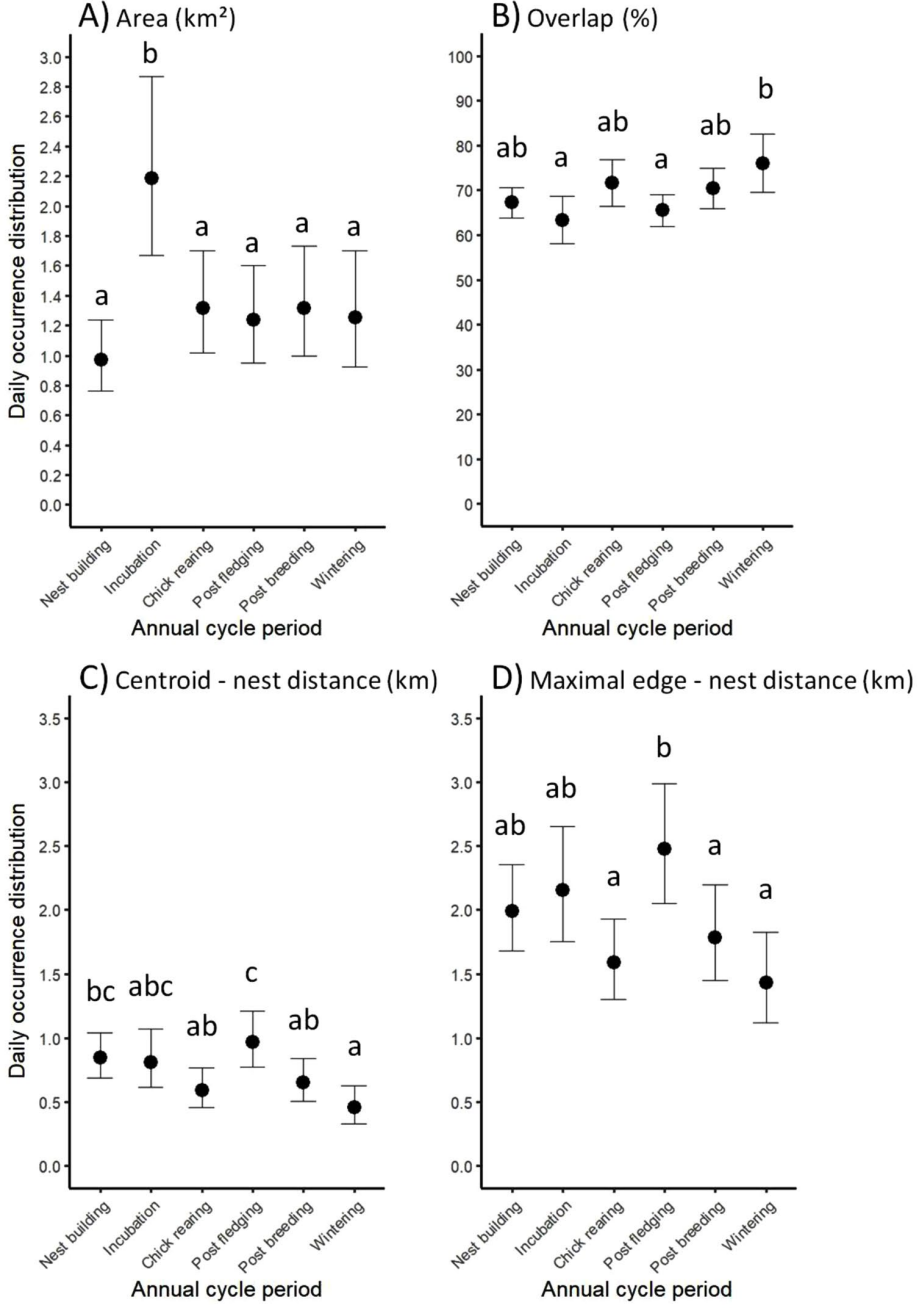
Variation in daily occurrence distribution according to the biological periods of jackdaws. The four descriptors used are (**A**) daily occurrence area (km^2^), (**B**) extent of the (intra-period) spatial overlap of the daily occurrence distributions (%), (**C**) distance between the daily occurrence centroid and the nest location (km), and (**D**) distance between the farthest limit of the daily occurrence distribution and the nest location (km). Mean estimates and 95% confidence intervals are provided. Different letters indicate statistically different values (increasing values arranged in alphabetical order)

Daily occurrence areas varied across periods (χ^2^ = 100.07, df = 5, *p* < 0.001, R^2^_m_ = 0.10, R^2^_c_ = 0.36; Table S3; Fig. 2A): they were higher (and highly variable among individuals) during incubation (2.19 km^2^ on average) compared to all other periods (0.97–1.32 km^2^). Daily occurrence distributions largely overlapped within each period, with the lowest mean values (63.39% and 65.49%) during incubation and post fledging, respectively, and a maximum mean value in winter (76.07%; χ^2^= 15.16, df = 5, *p* = 0.01, R^2^_m_ = 0.03, R^2^_c_ = 0.07; Table S3; Fig. 2B). Similarly, the distances from the nest location to the daily occurrence centroid and to the farthest boundary of the daily occurrence distribution varied across periods (χ^2^ = 36.86, df = 5, *p* < 0.001, R^2^_m_ = 0.07, R^2^_c_ = 0.19, and χ^2^ = 53.96, df = 5, *p* < 0.001, R^2^_m_ = 0.08, R^2^_c_ = 0.21, respectively; Table S3). Both distances were the shortest during chick rearing, post breeding and wintering, and the longest during post fledging (Fig. 2C, D).

### Foraging habitat use and selection patterns

Interestingly, almost all available tracking days involved at least one foraging occasion in grasslands (daily use probabilities ≥ 0.97; Tables S2 and 2). In contrast, the daily use probability varied significantly in cereal and maize crops through time (χ^2^ = 75.54, df = 5, *p* < 0.001, R^2^_m_ = 0.18, R^2^_c_ = 0.40, and χ^2^ = 68.18, df = 5, *p* < 0.001, R^2^_m_ = 0.28, R^2^_c_ = 0.44, respectively; Tables S3 and 2). In cereal crops, the daily use probability was the highest during post fledging (0.77 on average) compared to the other periods (0.10–0.33). In maize crops, the highest probability was found during incubation (0.89), then during nest building and wintering (0.42 and 0.54, respectively).

**Table 2:** Comparisons of the daily foraging probability between biological periods for each main agricultural habitat

Agricultural habitat	Daily use probability					
	Nest building	Incubation	Chick rearing	Post fledging	Post breeding	Wintering
Grasslands	0.99	0.98	1.00	0.97	1.00	1.00
	(0.96;1.00)	(0.90;1.00)	(1.00;1.00)	(0.91;0.99)	(1.00;1.00)	(1.00;1.00)
Cereals	0.26^ab^	0.33^b^	0.10^a^	0.77^c^	0.29^b^	0.25^ab^
	(0.15;0.42)	(0.17;0.55)	(0.04;0.22)	(0.61;0.87)	(0.15;0.48)	(0.11;0.48)
Maize	0.42^bc^	0.89^d^	0.18^ab^	0.08^a^	0.10^a^	0.54^cd^
	(0.28;0.58)	(0.75;0.96)	(0.09;0.35)	(0.04;0.16)	(0.04;0.23)	(0.30;0.77)

Mean estimates with 95% confidence intervals in brackets. No model was computed for grasslands since foraging occasions were recorded in almost all tracking days. Different letters indicate statistically different values (increasing values arranged in alphabetical order) for pairwise post-hoc comparisons across periods

**Fig. 3:**
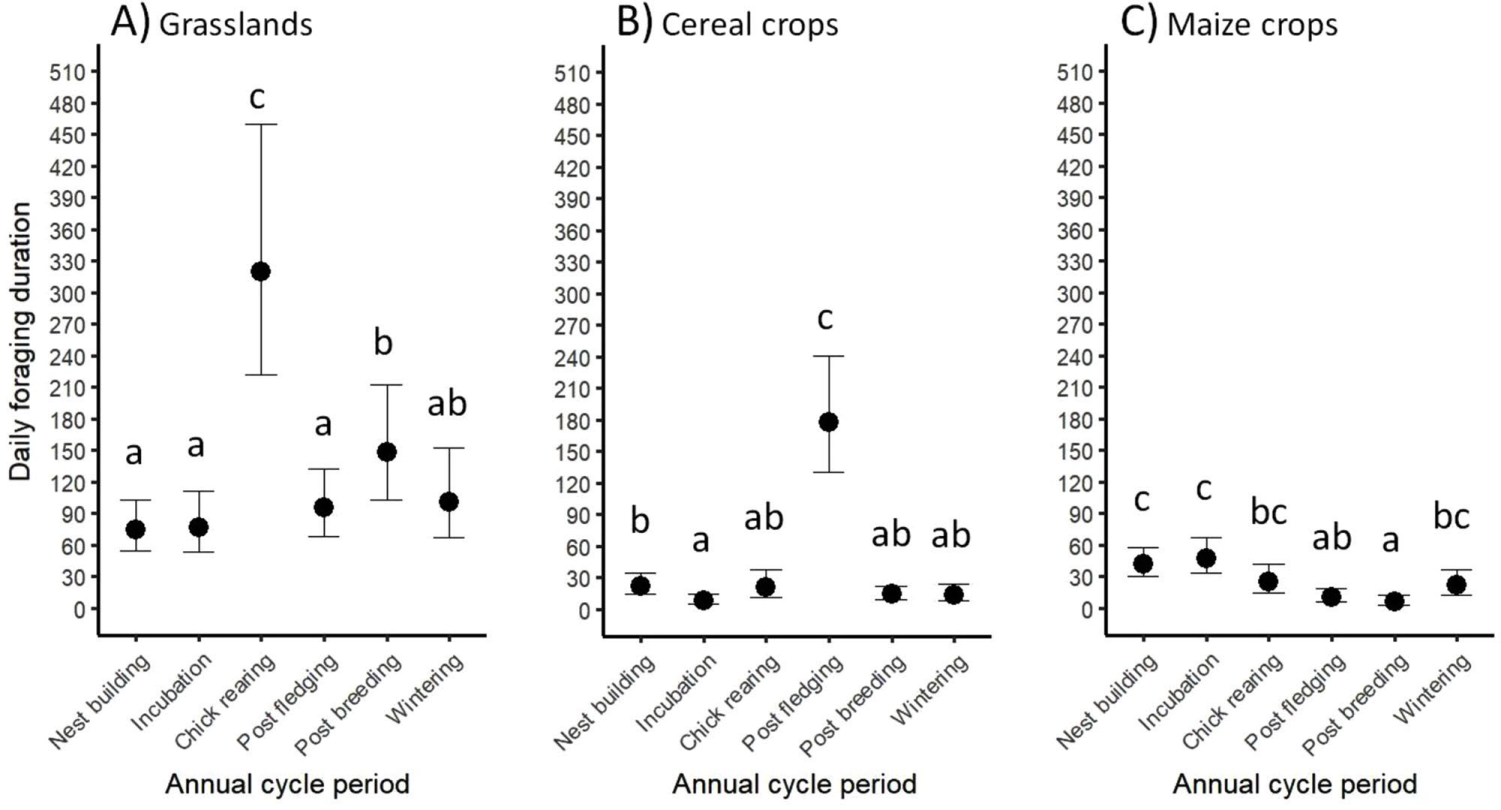
Variation in daily foraging duration spent by jackdaws in different habitats according to their biological periods. Habitats are (**A**) grasslands, (**B**) cereal crops, and (**C**) maize crops. Mean estimates (min) and 95% confidence intervals are provided. Different letters indicate statistically different values (increasing values arranged in alphabetical order)

The daily time (conditional on use) spent foraging varied significantly and unevenly in the three most visited habitats according to the annual periods, suggesting seasonal variation in food opportunities (37.32 < χ^2^ < 384.78, df = 5, *p* < 0.001, 0.20 < R^2^_m_ < 0.65, 0.41 < R^2^_c_ < 0.73; Table S3; Fig. 3). The time spent foraging in grasslands was significantly longer during chick rearing (320 min per day on average; Fig. 3A) compared to the other periods. Regarding cereals, it was the highest during post fledging (177 min per day; Fig. 3B). In maize fields, the longest foraging duration was during the first two stages of the breeding period (42 and then 47 min per day), and was moderate during chick-rearing and wintering (25 and then 22 min per day; Fig. 3C).

**Fig. 4:**
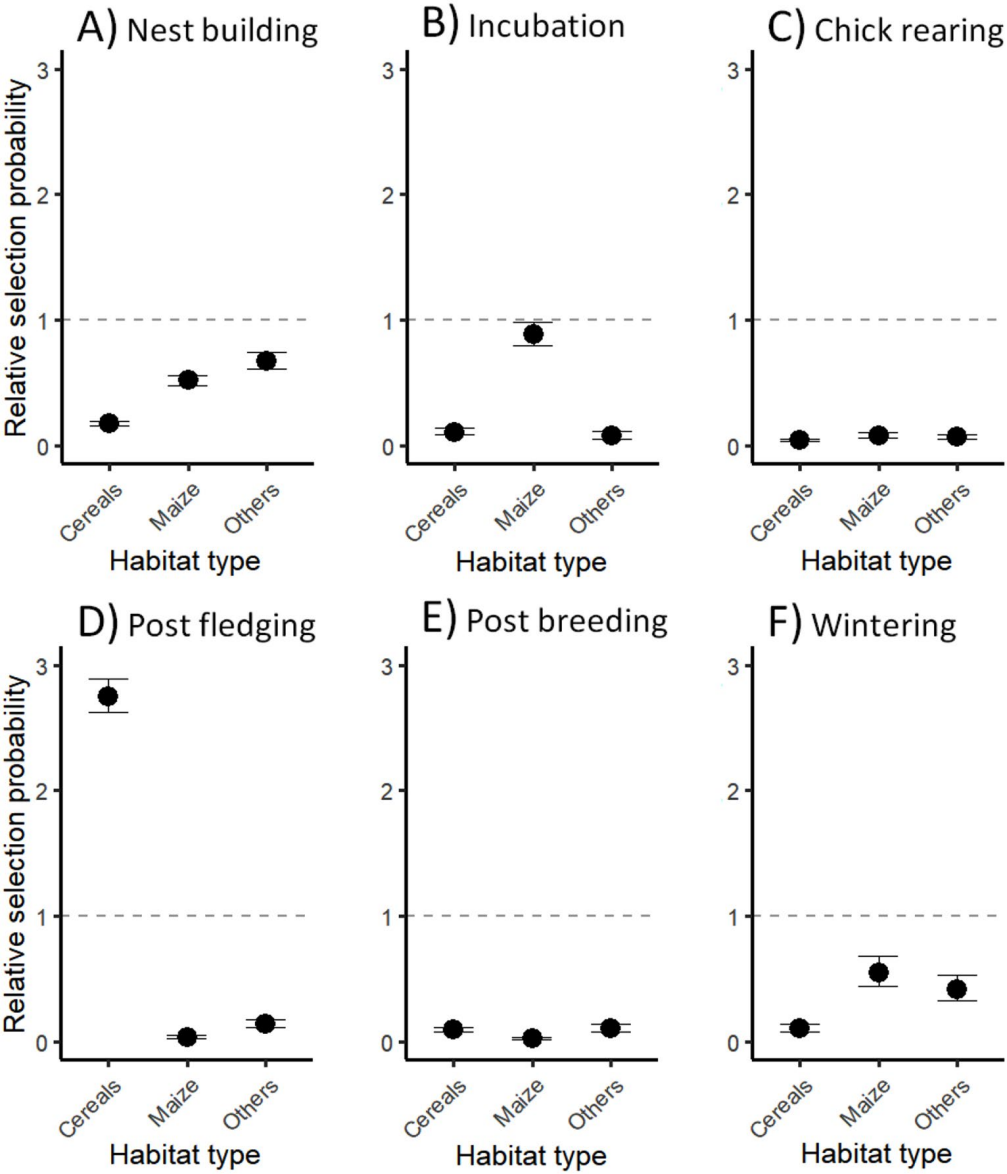
Relative selection strength estimates for foraging habitats by jackdaws throughout their annual life cycle. Biological periods are (**A**) nest building, (**B**) incubation, (**C**) chick rearing, (**D**) post fledging, (**E**) post breeding, and (**F**) wintering. Estimates (mean values with 95% confidence intervals; exponentiated coefficients) were compared to the selection strength computed for grasslands as a reference level (dashed line; all comparisons led to *p* < 0.001, except for maize during incubation). Values above and below the dashed line indicate higher and lower relative intensities of use, respectively, under equal habitat availability with grasslands

Jackdaws showed a strong preference for grasslands throughout the annual life-cycle, except during incubation when they equally selected maize fields and grasslands, and during post fledging when they preferentially selected cereal fields (Fig. 4; Table S4).

## Discussion

Productive crops in agricultural landscapes are highly attractive to a number of birds potentially causing substantial economic damage [8–10]. This issue requires utmost attention for sedentary birds that may impact agricultural crops at any stage of their annual life cycle. In the present work, we documented unique findings on the circum-annual movements of GPS-tagged adult male jackdaws in an agricultural region where bird-farmer conflicts are critical.

### Year-round fidelity to the close surroundings of the nesting place

Daily occurrence areas were restricted (1.0–1.3 km^2^ on average, but see below), relatively centered on the nesting places (0.43–0.85 km on average between the nest and the centroid of daily occurrence distributions), and the farthest daily-activity distances from the nests did not exceed 1.9 km (average value throughout the year). In turn, adult jackdaws moved around their nesting place– to which they are strongly faithful all year long [26]– during daytime, resulting in high spatial overlap in daily occurrence distributions (63–76% according to biological periods). To our knowledge, there is no comparable study to date in this species. Feeding distances of jackdaws have been documented in an earlier work conducted in an urban population exploiting agricultural food resources [35], and suggested that birds remain close to their nest. However, in this paper, no values are provided on occurrence areas and their stability over time [35]. Being faithful to a nesting locality as we showed can be beneficial in terms of (i) access to better-quality spots and socially acquired information on shared foraging grounds from conspecific neighboring breeders, and (ii) high familiarity in the use of the nearby environment ([59] and references therein, on the Common raven *Corvus corax*). High attachment with the close surroundings of the nests has indeed been found to strongly limit energy-expensive foraging flights and substantially increase successful food intake rates in good-quality fields (e.g. [60]).

The daily occurrence areas were larger during egg incubation (ca. 2.2 km^2^ on average) when males feed their mate [27, 44]. Increased food demands during this period are probably not the main reason for the increased daily occurrence area, which would otherwise also have been larger during chick rearing– a period of intensive foraging activity for male jackdaws [27]. In other studies, jackdaw foraging flights are not necessarily longer during periods of high food demand [26, 35]. An alternative explanation could be changes in food opportunities during this specific period as egg incubation overlapped with the maize sowing period (Fig. 1). During this critical period, jackdaws we studied actively explored farmed land to locate newly patchily sown maize fields and potentially shifted to this new food resource (see similar findings in other animals; [61, 62]). Additionally, it cannot be excluded that scaring actions deployed during germination and early seedling growth (propane cannons, tapes strung across fields and human bird scarers) increased the movements of jackdaws [63].

Jackdaws flew farther from their nest during the post-fledging period. This finding is consistent with the fact that breeders defend their nest less during the weeks following fledging (personal observations) and jointly spend time with the juveniles in feeding grounds [26, 64]. However, adults restart defending their nesting place from post breeding [26], which coincides with a drop in the length of the two distances from the nest we measured (Fig. 2C, D), meaning that competition for nest places is strong, notably in view of the growing jackdaw population size.

Finally, the relative temporal consistency of the occurrence distributions of breeding jackdaws suggests that sufficient food resources were available in agricultural land in the vicinity of the nesting places.

### Predominant role of grasslands as feeding grounds

Grasslands, cereal and maize crops altogether represented 92% of agricultural land in the study area, and were unsurprisingly the most visited foraging habitats regardless of the annual cycle period of jackdaws. Furthermore, jackdaws visited grasslands every day to forage (daily use probabilities ≥ 0.97), and spent on average a six-fold longer foraging time in grasslands than in cultivated areas, except during post fledging (see below). They even spend up to ten-fold longer foraging time in grasslands during chick rearing. Grasslands are rich in invertebrates [65, 66], a well-known key food resource during breeding for many birds including jackdaws [33, 67, 68]. Therefore, it is consistent that jackdaws foraged preferentially in grasslands during chick rearing, because grasslands probably offered higher foraging profitability than other agricultural habitats at that time of the year [69]. In addition, maize grain fragments are frequently found in cow dung in pastures [70], and likely constitute a supplement and attractive food resource for jackdaws at least for self-maintenance (feeding chicks with corn fragments remains to be explored). Arthropod items, and more specifically *Aphodius* spp. specimens generally found in cow dung, were very frequent in the diet of adult jackdaws (more details in Table S1). This validates the observed very frequent use of grasslands as feeding grounds during that period (but also during wintering). The role of grasslands as feeding grounds has been documented for other agricultural bird pests, for instance the European starling *Sturnus vulgaris* [66].

Cultivated crops are short-lived feeding grounds that opportunistic birds can extensively shift to during specific periods [71, 72]. In our study area, jackdaw-farmer conflicts mainly occur from sowing to the young plant stage of maize, and the pre-harvest period of cereals [8, 36, 37]. Daily foraging activities in maize crops were indeed higher during the peak of the sowing period (coinciding with nest building and incubation; Fig. 1) than during the rest of the annual life cycle. The daily foraging duration in maize crops during sowing was nevertheless moderate compared to grasslands. However, this finding does not necessarily mean that birds poorly used maize fields. The food intake / energy expenditure balance is probably high at that time since seeds are very accessible and allow fast intake rates (see similar findings in other birds in [7, 73]). An alternative explanation is that maize and other preferred foods (animal prey) were available in other habitats at that time (notably in grasslands as mentioned before), so that crops were comparatively less attractive and less exploited by jackdaws [8]. Furthermore, the intermediate foraging duration in maize crops in winter was likely linked to the seeds in harvested unplowed maize fields [70], in compliance with the importance of maize in the diet of adult jackdaws during this period (see Table S1).

Concerning cereals (wheat and barley crops), their use was the highest during post fledging, coinciding with the harvesting period peak, and this habitat type was indeed strongly selected at that time of the year. However, it remains unclear whether the birds exploited these fields during days preceding harvest (leading to depredation events) or just after harvest. Harvest may indeed provide a substantial quantity of grains fallen on the ground (which remains to be quantified), and facilitate foraging on soil invertebrates by dramatically reducing the field cover [23], leading to a newly attractive habitat.

In sum, jackdaws mainly feed in grasslands over their annual life cycle and adjust their resource use with additional short-lived cultivated food resources mainly found in maize and cereals crops, in line with their energy requirements for self-maintenance and reproduction [27]. Our results are to some extent comparable to those on the foraging habitat selection of agricultural areas by a bird of prey– the Barn owl *Tyto alba*– over its annual life cycle, in the sense that grasslands are strongly selected in such landscapes [18]. In our study, jackdaws were repeatedly confronted to unpredictable changes in agricultural habitats due to farming practices; therefore, they strongly selected for grasslands as feeding grounds during most of their annual life cycle and opportunistically fed on cultivated crops.

## Conclusive remarks, limitations and future prospects

As far as we know, our results provide the first insights into the daily movements and habitat use and selection by the commonly scorned Western jackdaw in agricultural areas throughout its annual life cycle. As central-place foragers, male adults were found to be very faithful to the close surroundings of their urban nesting place over the year. They preferentially used grasslands because they are likely simultaneously food-rich and food predictable grounds. However, the birds opportunistically fed on short-lived maize and cereal seeds, notably during the sowing and post-harvest periods, respectively, but not as dramatically as expected from damage declarations by farmers.

A question that remains unanswered is the potential effects of more intensively farmed landscapes on the foraging strategies of this bird. In other words, we wonder about (i) how jackdaws would respond to variable proportions of grassland areas and a decrease of invertebrate abundances [74], and (ii) what the ensuing crop damage would be. Therefore, we recommend that future studies take place across a range of agricultural landscapes to explore their influence on the habitat use pattern of this corvid, and possible ultimate consequences on its breeding performance [35].

One limitation of our study is that we focused on male adults, while habitat use by animals does not only depend on extrinsic factors but also on intrinsic ones. For example, younger individuals have been reported to move longer distances or show more nomadic behavior than breeders in a range of birds for a number of reasons [75–77]. Our tracking data of younger jackdaws also support this point (authors’ unpublished data, see also [26]). This means that our findings are partially indicative of agricultural habitat use by the Western jackdaw. Consequently, we call for further investigations to explore variation in space use by jackdaws according to sex and age to provide a broader understanding of environmental (for instance according to agricultural contexts) and intrinsic effects [78]. In the same vein, it would be informative in the near future to combine counts and food intake rate data across habitats with GPS-tracking to better assess the actual jackdaw depredation pressure on crops along the agricultural calendar. We hope that such future scientific studies will contribute to further improve jackdaw management strategies.

### Electronic supplementary material

Below is the link to the electronic supplementary material.

**Additional file 1: Table S1** Diet of jackdaws over the life-cycle periods. **Additional file 2: Fig. S1** Typical crop damage caused by jackdaws. **Additional file 3: Table S2** Details on the tracking days. **Additional file 4: Fig. S2** Example of agricultural land utilization. **Additional file 5: Table S3** Models explaining daily occurrence distributions and daily foraging probabilities and durations. **Additional file 6: Table S4** Models explaining foraging habitat selection

## Data Availability

The datasets used and/or analysed during the current study are available from the corresponding author on reasonable request.

## Abbreviations

GPS: Global Positioning System
GSM: Global System for Mobile
(G)LMM: (Generalized) Linear Mixed Model
HSF: Habitat Selection Function
