# Supplementary material for: Agricultural habitat use and selection by a sedentary bird over its annual life cycle in a crop-depredation context
Source: Mov Ecol. 2024 Mar 29;12:26. doi: 10.1186/s40462-024-00462-0 (PMC10981352; doi:10.1186/s40462-024-00462-0)
Supplement: Supplementary file 1 — Additional file 1: Table S1 Diet of jackdaws over the life-cycle periods. Additional file 2: Fig. S1 Typical crop damage caused by jackdaws. Additional file 3: Table S2 Details on the tracking days. Additional file 4: Fig. S2 Example of agricultural land utilization. Additional file 5: Table S3 Models explaining daily occurrence distributions and daily foraging probabilities and durations. Additional file 6: Table S4 Models explaining foraging habitat selection [file 40462_2024_462_MOESM1_ESM.docx]

# Additional File

**Table S1** Gizzard content of 80 adult male jackdaws across time

| Annual cycle period | Number of birds | Occurrence of food items (%) | | |
| --- | --- | --- | --- | --- |
|  |  | Maize | Wheat | Arthropods |
|  |  |  |  | (*Coleoptera*) |
| Nest construction | - | - | - | - |
| Incubation | 5 | 40 | 0 | 100 (40) |
| Chick rearing | 64 | 45 | 33 | 78 (59) |
| Post fledging | - | - | - | - |
| Post breeding | - | - | - | - |
| Wintering | 11 | 100 | 0 | 82 (18) |

Birds were collected opportunistically during licensed culling operations conducted in the Finistère district during three periods of their annual life cycle in 2020. The proportion of gizzards containing a given food item (occurrence) is provided for each biological period. Only maize, wheat and arthropods were considered for simplification, in line with the issues addressed in this work. The proportion of gizzards containing at least one arthropod specimen belonging to the *Coleoptera* order (mainly *Aphodius* spp – coprophagous beetles typically found in cow dung) is indicated in brackets. Overall, the provenance of maize and wheat is unknown and may depend on the annual cycle period (i.e. grain possibly stored, sown, pecked on mature plants before harvest, or present on the ground in the fields after harvest, plus present in cow dung for maize)


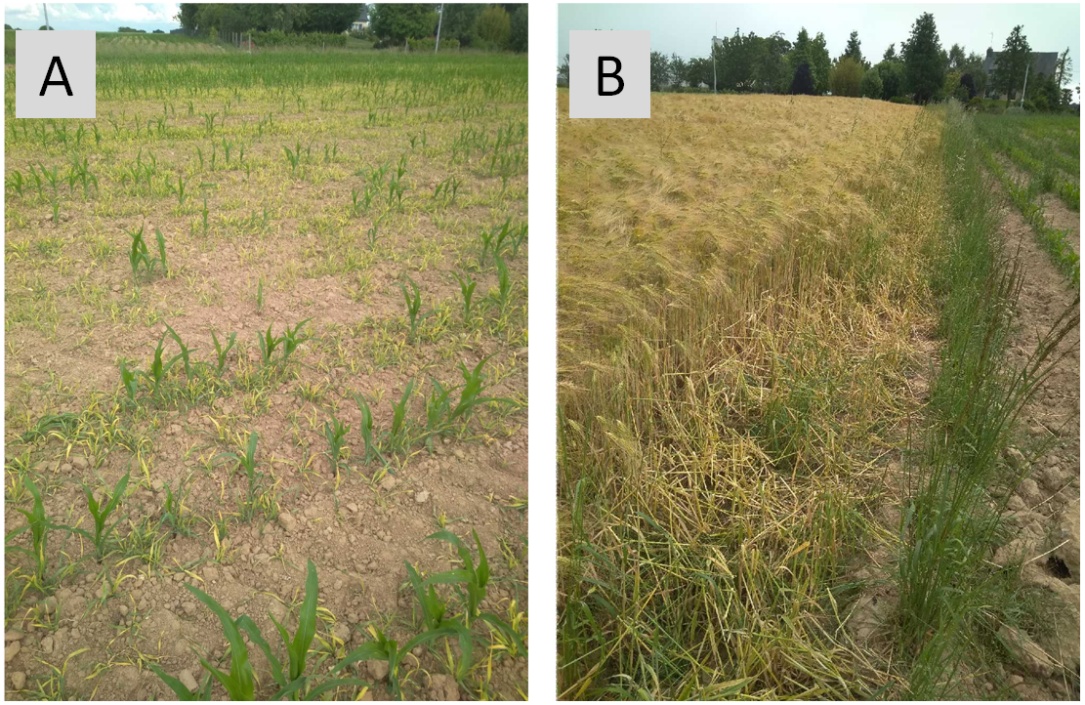


**Fig. S1** Typical crop damage caused by jackdaws in the study area. (A) On young maize plants. (B) On wheat ears before harvesting

**Table S2** Tracking days for each equipped bird over their annual life cycle in 2021 and 2022

|  |  |  | Number of tracking days | | | | | | | | | | | | | | |
| --- | --- | --- | --- | --- | --- | --- | --- | --- | --- | --- | --- | --- | --- | --- | --- | --- | --- |
| Age at  capture time | Nesting  town | Bird  identity | Total |  | Nest  building |  | Incubation |  | Chick  rearing | |  | Post  fledging | |  | Post  breeding |  | Wintering |
|  |  |  |  |  |  |  |  |  |  |  |  |  |  |  |  |  |  |
|  |  |  | 2021-2022 |  | 2022 |  | 2022 |  | 2021 | 2022 |  | 2021 | 2022 |  | 2021 |  | 2021 |
| Adult | R | 1 | 66 |  | 19 |  | 11 |  | 8 | 2 |  | 14 | - |  | 7 |  | 5 |
| Adult | R | 2 | 55 |  | 11 |  | 7 |  | 8 | 1 |  | 12 | - |  | 8 |  | 8 |
| Adult | R | 3 | 54 |  | 12 |  | 11 |  | 5 | - |  | 15 | 1 |  | 9 |  | 1 |
| Adult | R | 4 | 43 |  | 5 |  | 8 |  | 5 | - |  | 10 | - |  | 9 |  | 6 |
| Adult | R | 5 | 35 |  | 9 |  | 2 |  | 1 | - |  | 12 | - |  | 5 |  | 6 |
| Adult | R | 6 | 35 |  | 6 |  | - |  | 5 | 4 |  | 6 | 6 |  | 5 |  | 3 |
| Adult | R | 7 | 35 |  | 14 |  | - |  | 2 | - |  | 12 | - |  | 5 |  | 2 |
| Adult | R | 8 | 31 |  | 4 |  | 1 |  | 4 | - |  | 11 | 1 |  | 10 |  | - |
| Adult | R | 9 | 23 |  | 7 |  | 5 |  | 2 | - |  | 4 | - |  | 4 |  | 1 |
| Adult | R | 10 | 19 |  | - |  | - |  | 5 | - |  | 8 | - |  | 6 |  | - |
| Adult | R | 11 | 10 |  | - |  | - |  | 1 | - |  | 9 | - |  | - |  | - |
| Adult | M | 12 | 14 |  | 3 |  | 1 |  | 1 | - |  | 7 | - |  | 2 |  | - |
| Adult | Q | 13 | 43 |  | 13 |  | 4 |  | 3 | - |  | 10 | - |  | 7 |  | 6 |
| Immature | R | 14 | 12 |  | 9 |  | 2 |  | - | - |  | - | 1 |  | - |  | - |
| Immature | R | 15 | 1 |  | 1 |  | - |  | - | - |  | - | - |  | - |  | - |
| Immature | M | 16 | 19 |  | 14 |  | 3 |  | - | 2 |  | - | - |  | - |  | - |
| Immature | M | 17 | 9 |  | 8 |  | 1 |  | - | - |  | - | - |  | - |  | - |
| Immature | Q | 18 | 12 |  | 7 |  | 4 |  | - | 1 |  | - | - |  | - |  | - |
| Immature | Q | 19 | 4 |  | 4 |  | - |  | - | - |  | - | - |  | - |  | - |
| Immature | P | 20 | 12 |  | 10 |  | 1 |  | - | 1 |  | - | - |  | - |  | - |

The age at the time of the capture in 2021 (adult: > 1-year old; immature bird: 1-year old), and the nesting town (R: Riec-sur-Bélon, M: Moëlan-sur-Mer, Q: Quimperlé, and P: Plouay) are provided. All immature birds bred in 2022 as adults. A dash (-) indicates that no tracking day occurred. At least one foraging behavior occurred during each of these tracking days. The total number of tracking days was 532 (518, 237, and 184 with at least one foraging occasion in grasslands, cereal and maize fields, respectively)

**
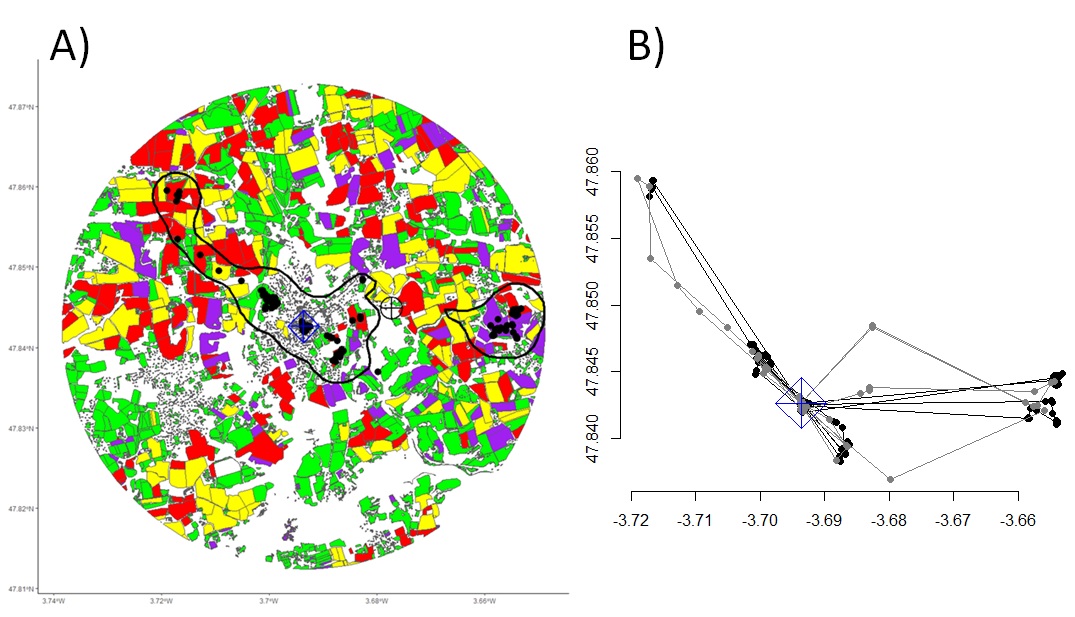
**

**Fig. S2** Example of agricultural land utilization by a tagged jackdaw during nest building. (A) 95% daily occurrence distribution (black line, edges; black dots, GPS points; calculated using a biased random bridge approach [1]) with its centroid (circle with a black border and a black cross inside), nest location (diamond with a blue border and a blue cross inside), and the main habitat types (green, yellow, red and purple polygons: grasslands, cereals, maize and other fields, respectively, urban areas being represented by small gray polygons). The buffer area (with a radius corresponding to the farthest distance between the limits of the daily occurrence distribution and the nest location) was used to generate random locations for modelling habitat selection. (B) GPS locations as flight and foraging occasions (black and gray connected dots, respectively) inferred from behavioral path-segmentation modeling [2]. Longitudes and latitudes (WGS 84 coordinate system) are provided in the X- and Y-axes, respectively, on both illustrations. Note that the proportion of each agricultural habitat available varied in each daily buffer area.

**Table S3** Parameter estimates of the annual life cycle period (6-level factor) explaining variation in each descriptor of the daily occurrence distributions and daily foraging probability and duration in the three main agricultural habitats utilized by jackdaws

| Response variable | Parameter estimates | | | | | | Statistics | | | |
| --- | --- | --- | --- | --- | --- | --- | --- | --- | --- | --- |
|  | Intercept | Incubation | Chick rearing | Post fledging | Post breeding | Wintering | ꭓ^2^_5_ | *p* | R²_m_ | R²_c_ |
|  |  |  |  |  |  |  |  |  |  |  |
| Daily occurrence distribution |  |  |  |  |  |  |  |  |  |  |
|  |  |  |  |  |  |  |  |  |  |  |
| Area (km²) | -0.03 | 0.81 | 0.30 | 0.24 | 0.30 | 0.26 | 100.07 | < 0.001 | 0.10 | 0.36 |
|  | (-0.27;0.21) | (0.65;0.97) | (0.04;0.56) | (-0.04;0.52) | (0.01;0.60) | (-0.07;0.58) |  |  |  |  |
|  |  |  |  |  |  |  |  |  |  |  |
| Overlap (%) | 67.21 | -3.83 | 4.45 | -1.73 | 3.24 | 8.85 | 15.16 | 0.01 | 0.03 | 0.07 |
|  | (63.81;70.62) | (-9.52;1.86) | (-1.69;10.59) | (-6.68;3.22) | (-2.44;8.92) | (1.58;16.13) |  |  |  |  |
|  |  |  |  |  |  |  |  |  |  |  |
| Centroid-nest | -0.16 | -0.04 | -0.36 | 0.13 | -0.26 | -0.62 | 36.86 | < 0.001 | 0.07 | 0.19 |
| distance (km) | (-0.37;0.04) | (-0.30;0.21) | (-0.65;-0.06) | (-0.15;0.42) | (-0.58;0.05) | (-0.99;-0.25) |  |  |  |  |
|  |  |  |  |  |  |  |  |  |  |  |
| Maximum edge- | 0.69 | 0.08 | -0.23 | 0.22 | -0.11 | -0.33 | 53.96 | < 0.001 | 0.08 | 0.21 |
| nest distance (km) | (0.52;0.86) | (-0.08;0.24) | (-0.45;-0.01) | (-0.01;0.45) | (-0.36;0.14) | (-0.61;0.05) |  |  |  |  |
|  |  |  |  |  |  |  |  |  |  |  |
| Daily foraging probability |  |  |  |  |  |  |  |  |  |  |
|  |  |  |  |  |  |  |  |  |  |  |
| Grasslands | - | - | - | - | - | - | - | - | - | - |
|  |  |  |  |  |  |  |  |  |  |  |
| Cereal fields | -1.03 | 0.32 | -1.18 | 2.23 | 0.13 | -0.06 | 75.54 | < 0.001 | 0.18 | 0.40 |
|  | (-1.74;-0.31) | (-0.50;1.14) | (-2.26;-0.10) | (1.27;3.18) | (-0.89;1.15) | (-1.22;1.11) |  |  |  |  |
|  |  |  |  |  |  |  |  |  |  |  |
| Maize fields | -0.31 | 2.40 | -1.18 | -2.15 | -1.84 | 0.47 | 68.18 | < 0.001 | 0.28 | 0.44 |
|  | (-0.95;0.33) | (1.50;3.30) | (-2.18;-0.18) | (-3.16;-1.15) | (-2.96;-0.72) | (-0.75;1.69) |  |  |  |  |
|  |  |  |  |  |  |  |  |  |  |  |
| Daily foraging duration (min) |  |  |  |  |  |  |  |  |  |  |
|  |  |  |  |  |  |  |  |  |  |  |
| Grasslands | 4.32 | 0.03 | 1.45 | 0.24 | 0.68 | 0.30 | 102.77 | < 0.001 | 0.20 | 0.46 |
|  | (4.00;4.64) | (-0.24;0.29) | (1.05;1.85) | (-0.13;0.61) | (0.27;1.09) | (-0.15;0.75) |  |  |  |  |
|  |  |  |  |  |  |  |  |  |  |  |
| Cereal fields | 3.10 | -0.94 | -0.07 | 2.07 | -0.41 | -0.46 | 384.78 | < 0.001 | 0.65 | 0.73 |
|  | (2.67;3.54) | (-1.43;-0.46) | (-0.78;0.65) | (1.55;2.60) | (-1.00;0.18) | (-1.15;0.24) |  |  |  |  |
|  |  |  |  |  |  |  |  |  |  |  |
| Maize fields | 3.74 | 0.11 | -0.52 | -1.36 | -1.84 | -0.66 | 37.32 | < 0.001 | 0.26 | 0.41 |
|  | (3.43;4.06) | (-0.23;0.45) | (-1.07;0.03) | (-1.97;-0.75) | (-2.52;-1.15) | (-1.27;-0.06) |  |  |  |  |
|  |  |  |  |  |  |  |  |  |  |  |

Mean estimates and 95% confidence intervals (in brackets) are provided. Nest building was used as the reference level (intercept). No model was computed for daily foraging probability in grasslands (-) since foraging occasions were recorded in almost all tracking days (see the text and **Table S2**)

**Table S4** Parameter estimates of the agricultural habitat (four-level factor) explaining variation in foraging habitat selection by jackdaws during each period of their annual life cycle

| Selection strength | Parameter estimates | | | Cross-correlation  coefficient |
| --- | --- | --- | --- | --- |
|  | Cereals | Maize | Others |  |
| Nest building | -1.76 | -0.66 | -0.40 | 0.60 |
|  | (-1.88;-1.65) | (-0.74;-0.59) | (-0.50;-0.30) |  |
| Incubation | -2.26 | -0.12 | -2.64 | 0.70 |
|  | (-2.52;-1.99) | (-0.23;-0.02) | (-3.07;-2.20) |  |
| Chick rearing | -3.19 | -2.55 | -2.74 | 0.67 |
|  | (-3.45;-2.93) | (-2.79;-2.32) | (-3.03;-2.45) |  |
| Post fledging | 1.01 | -3.36 | -1.97 | 0.56 |
|  | (0.96;1.06) | (-3.64;-3.07) | (-2.19;-1.75) |  |
| Post breeding | -2.38 | -3.94 | -2.30 | 0.95 |
|  | (-2.57;-2.19) | (-4.38;-3.50) | (-2.60;-1.99) |  |
| Wintering | -2.29 | -0.61 | -0.88 | 0.76 |
|  | (-2.62;-1.95) | (-0.83;-0.39) | (-1.13;-0.64) |  |

Mean estimates and 95% confidence intervals (in brackets) are provided. The habitat ‘others’ gathers the remaining agricultural habitat types. Grassland was used as the reference level, but this raw intercept estimate is not provided because it is irrelevant in habitat selection functions (fixed to 0 before calculating exponentiated and relative coefficients). The k-fold cross-correlation coefficient is provided for each model

**References**

1. Benhamou S, Cornelis D. Incorporating movement behavior and barriers to improve biological relevance of kernel home range space use estimates. J Wildlife Manage. 2010;74:1353–1360.

2. McClintock BT, Michelot T. momentuHMM: R package for generalized hidden Markov models of animal movement. Methods Ecol Evol. 2018;9:1518–1530.
